# A survey of rare coding variants in candidate genes in schizophrenia by deep sequencing

**DOI:** 10.1038/mp.2013.131

**Published:** 2013-10-15

**Authors:** X Hu, B Zhang, W Liu, S Paciga, W He, T A Lanz, R Kleiman, B Dougherty, S K Hall, A M McIntosh, S M Lawrie, A Power, S L John, D Blackwood, D St Clair, N J Brandon

**Affiliations:** 1 PharmaTherapeutics Precision Medicine, Pfizer Inc., Groton, CT, USA; 2 Research CoE, Groton, Pfizer Inc., Groton, CT, USA; 3 Research Statistics, Neuroscience, Pfizer Inc., Groton, CT, USA; 4 Neuroscience Research Unit, Pfizer Inc., Groton, CT, USA; 5 Division of Psychiatry, University of Edinburgh, Edinburgh, UK; 6 Department of applied medicine, University of Aberdeen, Aberdeen, UK

The genetic architecture of schizophrenia is likely contributed by both common and rare variants.^1^ Recent genome-wide studies have revealed that common variants in the major histocompatibility complex (MHC) region, *TCF4* and other genes are associated with schizophrenia.^1^ In addition, rare copy-number variation (CNV) regions in broad regions like 1q21.1, 15q13.3, 15q11.2, 22q11^1^ as well as individual genes such as *Neurexin*^2, 3^ have been identified. Unbiased exome or whole genome scanning procedures have the potential to identify novel loci while likely requiring large sample sets to reach a genome-wide significance level. It is possible that the previously identified genes/regions from high-throughput single-nucleotide polymorphisms (SNP) chip genome-wide scanning techniques, in contrast to some ‘classical' candidate genes,^4^ may harbor rare coding variants that have a role in disease risk. We selected a total of 101 genes from within the 1q21.1, 15q13.3, 22q11 and 15q11.2 regions and a number of other candidate genes, with either *a priori* knowledge for association with schizophrenia, for example, *TCF4/CCDC68, NRXN1*, or interesting for drug-discovery efforts, for example, cyclic nucleotide phosphodiesterase genes, and surveyed rare variants in their coding regions through deep sequencing.

Our sample set included cases who met DSM-IV criteria for schizophrenia. All subjects provided informed consent that was approved by the ethics committees at the specific sites. Our discovery set included 525 schizophrenia cases (68% male cases, 69 cases were diagnosed with schizophrenia before 18 years of age) and 619 controls (62% male cases) without any neurological and psychiatric disorders and were primarily collected during Pfizer clinical trials. The replication set includes 455 schizophrenia subjects (71% male subjects) and 336 controls (73.5% male subjects), collected at the Universities of Edinburgh and Aberdeen. Only Caucasian subjects were included in our study to reduce the sample heterogeneity.

Coding sequences in our target regions were enriched using the Nimblegen capture array, followed by Illumina HiSeq paired-end sequencing at the Beijing Genome Institute (BGI Inc.). We pooled 48 bar-coded samples together before the sequencing run. In total, we obtained 149 Mb of sequencing data in which over 98.5% of reads mapped to our regions of interest. The mean read depth was 96 × , which is much higher than the estimated average depth (33 × ) required for highly accurate downstream heterozygous variant detection. After removing genes with low coverage that failed the capture design, over 95.3% of the bases in our targeted regions were covered with genotype data at least 30 × to ensure variant detection sensitivity. The variants have a greater than 99.6% concordance rate with available genome-wide genotyping data.

A total of 7072 and 5170 novel variants were identified in the discovery and replication sets, respectively (we excluded all Indel calls, which may have a higher false-positive rate). Approximately, 70% of the variants are not common in the population (minor allele frequency number no greater than 1%). In both data sets, we found a variety of SNPs including intronic, missense, synonymous and UTR variants as well as splice variants and nonsense SNPs (Table 1). We observed approximately two fold rare (minor allele frequency number greater than 0.5%) nonsense alleles in cases compared with the nonsense alleles in controls (one-sided *P*-value=0.056, odd ratio (OR)=1.96). In contrast, we observed about equal frequencies of rare synonymous variants in cases and controls in the identical genomic regions for the same cohorts (one-sided *P*>0.1, OR=1.08), suggesting that it is unlikely that the result is due to sampling bias. Furthermore, the proportion of ultra-rare ‘deleterious' variants in the CNV regions is significantly higher in early-onset schizophrenia cases (age of onset less than 18 years) versus that in the controls in the study (nonsense plus splicing one-sided *P*-value=0.09, OR=3.41; including conserved damaging missense variants: one-sided *P*=0.02, OR=1.88), supporting the finding that rare variants may contribute to schizophrenia etiology. None of the rare nonsense variants identified in this study were listed in dbSNP (version 132). Intriguingly, different stop codons in *NRXN1* were observed in two individuals with schizophrenia from two independent cohorts but were not observed in any of the controls, suggesting that rare loss-of-function events in *NRXN1*, either through deletion or through nonsense mutation, could be important in the etiology of schizophrenia (Supplementary Table S1).

Most of the rare variants only occur once or twice in our cohort, which limits the statistical power to detect the association in individual variants. We therefore conducted aggregate analysis across all functional variants within each gene by comparing carrier frequencies between cases and controls to understand whether the gene as a whole has a consistent effect across the discovery and replication data sets. We focused on functional variants with a minor allele frequency no higher than 1% in controls in our analysis.

Among the 84 genes with at least one rare functional variant tested in both sample sets, 48 genes showed a consistent pattern of frequency distribution (Supplementary Table S2) although none of these associations passed the multiple test correction. Among these 48 genes, a majority of genes (30) showed an elevated frequency of rare variants in cases compared with controls, including the *TCF4* gene. Common SNPs in *TCF4* have emerged from the schizophrenia genome-wide association study (GWAS) consortia and confirmed to be associated, at genome-wide levels of significance, with the disease risk^1, 5^. Furthermore, one of the SNPs (rs9960767) has been linked to deficits in sensorimotor gating,^6^ and the expression levels of *TCF4* were shown to be increased in patients with psychosis^7^ and be under the regulation of the schizophrenia-linked miRNA-137.^8^ Rare mutations in *TCF4* have been previously identified in autosomal dominant forms of the Pitt–Hopkins syndrome, a disorder characterized by severe motor and mental retardation and susceptibility to childhood-onset seizures.^9^ A total of seven distinct rare functional variants in *TCF4* were identified in our two cohorts; intriguingly, they do not overlap with the known Pitt–Hopkins-associated variants (Supplementary Table S3). Three different variants were identified in the discovery cohort, with one variant (chr18:52928743:G_A) carried by three sporadic schizophrenia cases. Five variants occurred in the replication cohort and they all appeared in cases. The variant chr18:52928743:G_A is observed in a total of five schizophrenia cases and one control across the two cohorts. The same variant has a consistently rare frequency in the large general population (9/6494 from the Exome Variant server; 1/947 in our controls) and is lower than what we observed in the schizophrenia subjects (5/922). *TCF4* is a complex gene with multiple transcripts with variation in their N-termini.^10^ The C terminus is shared between variants with a conserved basic helix-loop-helix domain, which is critical for dimerization (homo-, hetero-), DNA binding at Eprussi box (E-box) sequences and transcriptional activation. Intriguingly, Pitt–Hopkins mutations congregate in these C-terminal domains and have been shown to differentially impact these functions. The mutations we have identified are principally in the N-terminal domains, and depending on the different exons spliced into a specific transcript these may have impact on processes such as subcellular localization as well as protein–protein and protein–DNA interactions. Although beyond the scope of this work, it will be important to understand the functional impact of these identified variants in the context of transcripts expressed in the schizophrenic brain.

In summary, the study suggests that the current candidate genes obtained from unbiased GWAS and CNV scanning reports do harbor rare functional variants in sporadic schizophrenia patients. We observed an overall enrichment for damaging variants, especially nonsense variants. In particular, a similar effect was observed in early-onset cases. Together, this supports our hypothesis that rare coding (for example, loss of function) variants in deletion/SNP regions from previous genome-wide scanning reports may also contribute to the genetic architecture of schizophrenia. The sample sizes in the study limit our ability to pinpoint specific genes/variants but the identified variants, especially in *NXRN1* and *TCF4*, will be helpful in future functional genomic investigations of the genes and related biological pathways.

## Figures and Tables

**Table 1:** Variants identified in the two independent cohorts

	*Discovery (case=525, control=619)*						*Replication (case=456, control=336)*					
*Variant type*^a^	*Novel*	*dbSNP*	*MAF⩽1%*^b^	*Case only*	*Control only*	*Case and control*	*Novel*	*dbSNP*	*MAF⩽1%*^b^	*Case only*	*Control only*	*Case and control*
***Downstream***	72	57	92	27	39	63	69	56	80	32	27	66
***Intergenic***	64	49	89	39	30	44	54	29	63	30	17	36
***Intronic***	4001	3143	4967	1706	2066	3372	2953	2846	3512	1540	1130	3129
***ncRNA***	303	258	350	129	129	303	236	228	264	111	75	278
***Nonsynonymous***	812	241	918	323	412	318	596	193	642	304	203	281
***Splicing***	31	11	33	12	18	12	18	8	17	10	4	12
***Stop codon***	16	3	15	9	5	5	11	3	10	7	1	6
***Synonymous***	600	305	733	246	320	339	381	247	452	215	131	282
***Upstream***	67	46	77	25	35	53	74	51	82	36	32	57
***UTR3***	925	425	1067	370	415	565	649	360	719	320	228	461
***UTR5***	181	59	203	47	97	96	130	56	145	67	37	82

Abbreviations: dbSNP, single-nucleotide polymorphism database; MAF, minor allele frequency.

a Transcripts from ENSEMBL V63 were used to annotate these variants.

b MAF less than or equal to 1% in each of the cohorts.

## Notes

XH, BZ, WL, SP, WH, TL, RK, BD, SH, AP, SLJ, and NJB are/were employees of Pfizer. The authors declare no conflict of interest.

## References

[bib1] Sullivan PF Daly MJ O'Donovan M Nat Rev Genet 2012 13 537 22777127 10.1038/nrg3240 PMC4110909

[bib2] Rujescu D Ingason A Cichon S Pietiläinen OP Barnes MR Toulopoulou T Hum Mol Genet 2009 18 988 18945720 10.1093/hmg/ddn351 PMC2695245

[bib3] Kirov G Rujescu D Ingason A Collier DA O'Donovan MC Owen MJ Schizophr Bull 2009 35 851 19675094 10.1093/schbul/sbp079 PMC2728827

[bib4] Crowley JJ Mol Psychiatry 2012 18 138 22472875 10.1038/mp.2012.28 PMC3577417

[bib5] Stefansson H Ophoff RA Steinberg S Andreassen OA Cichon S Rujescu D Nature 2009 460 744 19571808 10.1038/nature08186 PMC3077530

[bib6] Quednow BB Ettinger U Mössner R Rujescu D Giegling I Collier DA J Neurosci 2011 31 6684 21543597 10.1523/JNEUROSCI.0526-11.2011 PMC6632862

[bib7] Wirgenes KV Sønderby IE Haukvik UK Mattingsdal M Tesli M Athanasiu L Transl Psychiatry 2012 2 e112 22832956 10.1038/tp.2012.39 PMC3365258

[bib8] Kwon E Wang W Tsai LH Mol Psychiatry 2011 18 11 22182936 10.1038/mp.2011.170

[bib9] Blake DJ Forrest M Chapman RM Tinsley CL O'Donovan MC Owen MJ Schizophr Bull 2010 36 443 20421335 10.1093/schbul/sbq035 PMC2879683

[bib10] Sepp M Kannike K Eesmaa A Urb M. Timmusk T PLoS One 2011 6 e22138 21789225 10.1371/journal.pone.0022138 PMC3137626

