# Supplementary material for: A survey of rare coding variants in candidate genes in schizophrenia by deep sequencing
Source: Mol Psychiatry. 2013 Oct 15;19(8):858–9. doi: 10.1038/mp.2013.131 (PMC4113932; doi:10.1038/mp.2013.131)
Supplement: Supplementary Tables [file mp2013131x1.doc]

| Table S1. List of rare (minor allele frequency no greater than 0.5%) nonsense variants in the two independent sets. | | | | | | |  |  |  |  |  |  |  |  |  |
| --- | --- | --- | --- | --- | --- | --- | --- | --- | --- | --- | --- | --- | --- | --- | --- |
| *Set* | *Cat.* | *chr* | *start* | *ref* | *mut* | *gene* | *Stop Codon* | *# Major Homo. Cases* | *# Major Homo. Controls* | *# Het. Case* | *# Het. Control* | *# Minor Homo. Cases* | *# Minor Homo. Controls* |  | |
| Discovery | CaseOnly | 1 | 146684060 | A | C | FMO5 | gain | 523 | 619 | 2 | 0 | 0 | 0 |  | |
| Discovery | CaseOnly | 15 | 31323249 | G | A | TRPM1 | gain | 524 | 619 | 1 | 0 | 0 | 0 |  | |
| Discovery | CaseOnly | 2 | 50850713 | G | C | NRXN1 | gain | 524 | 619 | 1 | 0 | 0 | 0 |  | |
| Discovery | CaseOnly | 22 | 18900990 | G | A | PRODH | gain | 524 | 619 | 1 | 0 | 0 | 0 |  | |
| Discovery | CaseOnly | 22 | 19137400 | C | A | GSC2 | gain | 234 | 199 | 1 | 0 | 0 | 0 |  | |
| Discovery | CaseOnly | 22 | 19221094 | G | A | CLTCL1 | gain | 524 | 619 | 1 | 0 | 0 | 0 |  | |
| Discovery | CaseOnly | 22 | 19486658 | C | T | CDC45 | gain | 524 | 619 | 1 | 0 | 0 | 0 |  | |
| Discovery | CaseOnly | 22 | 20194584 | C | T | LOC150197 | gain | 524 | 619 | 1 | 0 | 0 | 0 |  | |
| Discovery | CaseOnly | 22 | 20795962 | C | T | KLHL22 | gain | 524 | 619 | 1 | 0 | 0 | 0 |  | |
| Discovery | ControlOnly | 1 | 146656174 | A | T | FMO5 | gain | 525 | 617 | 0 | 2 | 0 | 0 |  | |
| Discovery | ControlOnly | 15 | 22956428 | G | A | CYFIP1 | gain | 525 | 618 | 0 | 1 | 0 | 0 |  | |
| Discovery | ControlOnly | 22 | 19349417 | G | A | HIRA | gain | 525 | 618 | 0 | 1 | 0 | 0 |  | |
| Discovery | ControlOnly | 22 | 20074162 | C | T | DGCR8 | gain | 525 | 618 | 0 | 1 | 0 | 0 |  | |
| Discovery | ControlOnly | 22 | 20760319 | C | G | ZNF74 | gain | 525 | 617 | 0 | 2 | 0 | 0 |  | |
| Replication | CaseOnly | 1 | 147415652 | C | T | GPR89B | gain | 452 | 336 | 1 | 0 | 0 | 0 |  | |
| Replication | CaseOnly | 2 | 51254805 | T | A | NRXN1 | gain | 452 | 336 | 1 | 0 | 0 | 0 |  | |
| Replication | CaseOnly | 22 | 20101054 | G | A | TRMT2A | gain | 452 | 336 | 1 | 0 | 0 | 0 |  | |
| Replication | CaseOnly | 22 | 21044859 | C | T | POM121L4P | gain | 446 | 332 | 0 | 0 | 1 | 0 |  | |
| Replication | CaseOnly | 22 | 21330032 | C | T | AIFM3 | gain | 452 | 336 | 1 | 0 | 0 | 0 |  | |
| Replication | CaseOnly | 22 | 21332042 | C | T | AIFM3 | gain | 452 | 336 | 1 | 0 | 0 | 0 |  | |
| Replication | CaseOnly | 9 | 87563521 | A | T | NTRK2 | gain | 452 | 336 | 1 | 0 | 0 | 0 |  | |
| Replication | ControlOnly | 1 | 147230443 | G | A | GJA5 | gain | 453 | 335 | 0 | 1 | 0 | 0 |  | |
| Replication | Both | 18 | 52609920 | C | A | CCDC68 | gain | 451 | 334 | 2 | 2 | 0 | 0 |  | |

| Table S2. Rare functional variants in 84 genes of interests in two independent cohorts. | | | | | | | | | | | | |  | |
| --- | --- | --- | --- | --- | --- | --- | --- | --- | --- | --- | --- | --- | --- | --- |
| ***Genes*** | ***Discovery*** | | | | | | ***Replication*** | | | | | | | ***OR Consistent*** |
|  | *OR* | *Cases ( Mutation Non-Carriers)* | *Cases (Mutation Carriers)* | *Controls (Mutation Non-Carriers)* | *Controls (Mutation Carriers)* | *Nominal P-value* | *OR* | *Cases (Mutation Non-Carriers)* | *Cases (Mutation Carriers)* | *Controls (Mutation Non-Carriers)* | *Controls (Mutation Carriers)* | *Nominal P-value* | |  |
| *AADAT* | *.* | *509* | *4* | *611* | *0* | *0.04312* | *0.74257* | *404* | *5* | *300* | *5* | *0.751289* | | *FALSE* |
| *ACP6* | *2.01473* | *498* | *15* | *602* | *9* | *0.10141* | *0.61675* | *404* | *5* | *299* | *6* | *0.542328* | | *FALSE* |
| *AIFM3* | *1.07455* | *495* | *18* | *591* | *20* | *0.86932* | *0.79133* | *394* | *15* | *291* | *14* | *0.56902* | | *FALSE* |
| *ARVCF* | *1.01527* | *490* | *23* | *584* | *27* | *1* | *1.29573* | *385* | *24* | *291* | *14* | *0.503211* | | *TRUE* |
| *BCL9* | *1.28432* | *479* | *34* | *579* | *32* | *0.3729* | *1.35806* | *391* | *18* | *295* | *10* | *0.559768* | | *TRUE* |
| *C22orf25* | *1.40617* | *492* | *21* | *593* | *18* | *0.32822* | *0.64646* | *402* | *7* | *297* | *8* | *0.43732* | | *FALSE* |
| *C22orf29* | *2.0611* | *491* | *22* | *598* | *13* | *0.03995* | *0.74194* | *403* | *6* | *299* | *6* | *0.770098* | | *FALSE* |
| *C22orf39* | *0.29639* | *512* | *1* | *607* | *4* | *0.38339* | *0.37132* | *408* | *1* | *303* | *2* | *0.578753* | | *TRUE* |
| *CCDC68* | *1.19253* | *509* | *4* | *607* | *4* | *1* | *2.00831* | *401* | *8* | *302* | *3* | *0.368608* | | *TRUE* |
| *CDC45* | *0.92693* | *488* | *25* | *579* | *32* | *0.8916* | *1.677* | *387* | *22* | *295* | *10* | *0.203807* | | *FALSE* |
| *CDH10* | *0.7924* | *509* | *4* | *605* | *6* | *0.76212* | *3.00247* | *405* | *4* | *304* | *1* | *0.399629* | | *FALSE* |
| *CDH8* | *1.34598* | *504* | *9* | *603* | *8* | *0.62694* | *1.19701* | *401* | *8* | *300* | *5* | *1* | | *TRUE* |
| *CDH9* | *1.196* | *500* | *13* | *598* | *13* | *0.69355* | *1.5063* | *397* | *12* | *299* | *6* | *0.476926* | | *TRUE* |
| *CHD1L* | *1.19919* | *492* | *21* | *590* | *21* | *0.63655* | *1.15483* | *389* | *20* | *292* | *13* | *0.722842* | | *TRUE* |
| *CLDN5* | *0.23672* | *512* | *1* | *606* | *5* | *0.22819* | *0.93131* | *404* | *5* | *301* | *4* | *1* | | *TRUE* |
| *CLTCL1* | *0.95381* | *472* | *41* | *560* | *51* | *0.9131* | *0.88654* | *379* | *30* | *280* | *25* | *0.673052* | | *TRUE* |
| *CNTNAP2* | *1.10871* | *500* | *13* | *597* | *14* | *0.84631* | *0.73604* | *394* | *15* | *290* | *15* | *0.453336* | | *FALSE* |
| *COMT* | *1.67668* | *506* | *7* | *606* | *5* | *0.39842* | *.* | *406* | *3* | *305* | *0* | *0.264883* | | *TRUE* |
| *CRKL* | *1.19216* | *510* | *3* | *608* | *3* | *1* | *.* | *405* | *4* | *305* | *0* | *0.139918* | | *TRUE* |
| *CYFIP1* | *0.74534* | *483* | *30* | *564* | *47* | *0.23759* | *1.4263* | *392* | *17* | *296* | *9* | *0.427125* | | *FALSE* |
| *DGCR14* | *1.47793* | *479* | *34* | *583* | *28* | *0.1497* | *1.03404* | *391* | *18* | *292* | *13* | *1* | | *TRUE* |
| *DGCR2* | *2.0979* | *494* | *19* | *600* | *11* | *0.06218* | *1.24896* | *399* | *10* | *299* | *6* | *0.800576* | | *TRUE* |
| *DGCR6* | *1.08015* | *485* | *28* | *580* | *31* | *0.78966* | *2.78224* | *398* | *11* | *302* | *3* | *0.170417* | | *TRUE* |
| *DGCR6L* | *.* | *513* | *0* | *609* | *2* | *0.50336* | *.* | *409* | *0* | *302* | *3* | *0.077508* | | *TRUE* |
| *DGCR8* | *0.61563* | *502* | *11* | *590* | *21* | *0.212* | *1.95981* | *391* | *18* | *298* | *7* | *0.15234* | | *FALSE* |
| *FAN1* | *1.11937* | *472* | *41* | *567* | *44* | *0.65134* | *1.1231* | *394* | *15* | *295* | *10* | *0.839412* | | *TRUE* |
| *FMO5* | *0.92964* | *495* | *18* | *588* | *23* | *0.87402* | *1.20519* | *385* | *24* | *290* | *15* | *0.621054* | | *FALSE* |
| *GABRA2* | *.* | *512* | *1* | *611* | *0* | *0.45641* | *.* | *408* | *1* | *305* | *0* | *1* | | *TRUE* |
| *GABRB3* | *0.59154* | *508* | *5* | *601* | *10* | *0.43706* | *3.00247* | *405* | *4* | *304* | *1* | *0.399629* | | *FALSE* |
| *GABRG3* | *0.59314* | *510* | *3* | *605* | *6* | *0.52098* | *.* | *407* | *2* | *305* | *0* | *0.509922* | | *FALSE* |
| *GJA5* | *1.79118* | *510* | *3* | *609* | *2* | *0.66472* | *1.11946* | *406* | *3* | *303* | *2* | *1* | | *TRUE* |
| *GJA8* | *1.40047* | *499* | *14* | *599* | *12* | *0.43016* | *0.36667* | *405* | *4* | *297* | *8* | *0.138614* | | *FALSE* |
| *GNB1L* | *0.49131* | *508* | *5* | *599* | *12* | *0.22278* | *.* | *406* | *3* | *305* | *0* | *0.264883* | | *FALSE* |
| *GRM2* | *0.41351* | *507* | *6* | *594* | *17* | *0.06022* | *1.1766* | *398* | *11* | *298* | *7* | *0.813106* | | *FALSE* |
| *GRM3* | *0.29639* | *512* | *1* | *607* | *4* | *0.38339* | *.* | *409* | *0* | *304* | *1* | *0.427171* | | *TRUE* |
| *HIC2* | *1.19483* | *503* | *10* | *601* | *10* | *0.82165* | *1.12035* | *403* | *6* | *301* | *4* | *1* | | *TRUE* |
| *HIRA* | *0.87065* | *502* | *11* | *596* | *15* | *0.84296* | *0.51368* | *402* | *7* | *295* | *10* | *0.216373* | | *TRUE* |
| *HTR2C* | *.* | *513* | *0* | *610* | *1* | *1* | *0.49468* | *407* | *2* | *302* | *3* | *0.655844* | | *TRUE* |
| *KLHL22* | *0.33771* | *511* | *2* | *604* | *7* | *0.19289* | *0.74384* | *406* | *3* | *302* | *3* | *0.704385* | | *TRUE* |
| *LOC150197* | *1.39493* | *506* | *7* | *605* | *6* | *0.58606* | *0.36978* | *407* | *2* | *301* | *4* | *0.410226* | | *FALSE* |
| *LOC400891* | *0.93849* | *498* | *15* | *592* | *19* | *1* | *1.1766* | *398* | *11* | *298* | *7* | *0.813106* | | *FALSE* |
| *LZTR1* | *1.3669* | *505* | *8* | *604* | *7* | *0.60776* | *1.04478* | *402* | *7* | *300* | *5* | *1* | | *TRUE* |
| *MAGEL2* | *0.97355* | *495* | *18* | *589* | *22* | *1* | *1.41339* | *394* | *15* | *297* | *8* | *0.523287* | | *FALSE* |
| *MED15* | *1.71085* | *496* | *17* | *599* | *12* | *0.18675* | *1.31032* | *402* | *7* | *301* | *4* | *0.766057* | | *TRUE* |
| *MKRN3* | *0.47436* | *511* | *2* | *606* | *5* | *0.46411* | *0.37132* | *408* | *1* | *303* | *2* | *0.578753* | | *TRUE* |
| *MRPL40* | *0.71294* | *510* | *3* | *606* | *5* | *0.73388* | *3.00247* | *405* | *4* | *304* | *1* | *0.399629* | | *FALSE* |
| *MTMR10* | *0.61234* | *501* | *12* | *588* | *23* | *0.22714* | *0.91446* | *393* | *16* | *292* | *13* | *0.84951* | | *TRUE* |
| *NDN* | *.* | *513* | *0* | *610* | *1* | *1* | *.* | *365* | *1* | *293* | *0* | *1* | | *FALSE* |
| *NIPA1* | *.* | *513* | *0* | *610* | *1* | *1* | *1.11946* | *406* | *3* | *303* | *2* | *1* | | *FALSE* |
| *NIPA2* | *0.59393* | *511* | *2* | *607* | *4* | *0.69378* | *6.84* | *400* | *9* | *304* | *1* | *0.049897* | | *FALSE* |
| *NLGN4X* | *0.47436* | *511* | *2* | *606* | *5* | *0.46411* | *.* | *407* | *2* | *305* | *0* | *0.509922* | | *FALSE* |
| *NRXN1* | *0.466* | *503* | *10* | *586* | *25* | *0.05643* | *0.87997* | *390* | *19* | *289* | *16* | *0.728883* | | *TRUE* |
| *NTRK2* | *1.87664* | *499* | *14* | *602* | *9* | *0.14548* | *2.16114* | *392* | *17* | *299* | *6* | *0.133267* | | *TRUE* |
| *OTUD7A* | *.* | *510* | *3* | *611* | *0* | *0.09477* | *0.74447* | *407* | *2* | *303* | *2* | *1* | | *FALSE* |
| *P2RX6* | *1.05963* | *505* | *8* | *602* | *9* | *1* | *1.50886* | *395* | *14* | *298* | *7* | *0.502993* | | *TRUE* |
| *PDE10A* | *1.19406* | *505* | *8* | *603* | *8* | *0.80304* | *0.84931* | *401* | *8* | *298* | *7* | *0.796032* | | *FALSE* |
| *PDE2A* | *2.09931* | *506* | *7* | *607* | *4* | *0.24321* | *1.4963* | *405* | *4* | *303* | *2* | *1* | | *TRUE* |
| *PDE4B* | *2.99705* | *508* | *5* | *609* | *2* | *0.25641* | *2.63806* | *402* | *7* | *303* | *2* | *0.313816* | | *TRUE* |
| *PDE8A* | *1.31693* | *502* | *11* | *601* | *10* | *0.6594* | *0.84931* | *401* | *8* | *298* | *7* | *0.796032* | | *FALSE* |
| *PDE8B* | *1.4936* | *508* | *5* | *607* | *4* | *0.73946* | *0.74384* | *406* | *3* | *302* | *3* | *0.704385* | | *FALSE* |
| *PDE9A* | *1.49139* | *492* | *21* | *594* | *17* | *0.2485* | *0.74321* | *405* | *4* | *301* | *4* | *0.729437* | | *FALSE* |
| *PI4KA* | *0.64751* | *502* | *11* | *591* | *20* | *0.27694* | *1.06695* | *399* | *10* | *298* | *7* | *1* | | *FALSE* |
| *PRKAB2* | *0.39583* | *512* | *1* | *608* | *3* | *0.63003* | *0.7451* | *408* | *1* | *304* | *1* | *1* | | *TRUE* |
| *PRODH* | *0.75527* | *478* | *35* | *557* | *54* | *0.22418* | *1.47544* | *380* | *29* | *290* | *15* | *0.272018* | | *FALSE* |
| *RANBP1* | *2.38748* | *511* | *2* | *610* | *1* | *0.59499* | *.* | *406* | *3* | *305* | *0* | *0.264883* | | *TRUE* |
| *RAPGEF4* | *1.19522* | *502* | *11* | *600* | *11* | *0.82949* | *1.84621* | *392* | *17* | *298* | *7* | *0.210152* | | *TRUE* |
| *RTN4R* | *.* | *513* | *0* | *609* | *2* | *0.50336* | *.* | *406* | *3* | *305* | *0* | *0.264883* | | *FALSE* |
| *SERPIND1* | *1.20041* | *489* | *24* | *587* | *24* | *0.55641* | *1.12035* | *403* | *6* | *301* | *4* | *1* | | *TRUE* |
| *SLC25A1* | *0.59473* | *512* | *1* | *609* | *2* | *1* | *0.74447* | *407* | *2* | *303* | *2* | *1* | | *TRUE* |
| *SLC7A4* | *1.20206* | *485* | *28* | *583* | *28* | *0.58237* | *1.25868* | *384* | *25* | *290* | *15* | *0.515928* | | *TRUE* |
| *SNAP29* | *1.19406* | *505* | *8* | *603* | *8* | *0.80304* | *1.49386* | *407* | *2* | *304* | *1* | *1* | | *TRUE* |
| *SNRPN* | *1.31693* | *502* | *11* | *601* | *10* | *0.6594* | *.* | *409* | *0* | *304* | *1* | *0.427171* | | *FALSE* |
| *SNURF* | *1.59267* | *509* | *4* | *608* | *3* | *0.70857* | *.* | *408* | *1* | *305* | *0* | *1* | | *TRUE* |
| *TCF4* | *1.19216* | *510* | *3* | *608* | *3* | *1* | *.* | *403* | *6* | *305* | *0* | *0.040683* | | *TRUE* |
| *THAP7* | *1.19483* | *503* | *10* | *601* | *10* | *0.82165* | *1.24587* | *404* | *5* | *302* | *3* | *1* | | *TRUE* |
| *TRMT2A* | *0.94071* | *494* | *19* | *587* | *24* | *0.87719* | *0.79133* | *394* | *15* | *291* | *14* | *0.56902* | | *TRUE* |
| *TRPM1* | *0.93277* | *476* | *37* | *564* | *47* | *0.82018* | *0.72703* | *381* | *28* | *277* | *28* | *0.262922* | | *TRUE* |
| *TSSK2* | *0.93441* | *502* | *11* | *597* | *14* | *1* | *0.42046* | *405* | *4* | *298* | *7* | *0.219703* | | *TRUE* |
| *TUBGCP5* | *1.48737* | *486* | *27* | *589* | *22* | *0.18869* | *0.93045* | *399* | *10* | *297* | *8* | *1* | | *FALSE* |
| *TXNRD2* | *1.30578* | *487* | *26* | *587* | *24* | *0.3854* | *0.93045* | *399* | *10* | *297* | *8* | *1* | | *FALSE* |
| *UBE3A* | *.* | *513* | *0* | *607* | *4* | *0.13004* | *0.55603* | *406* | *3* | *301* | *4* | *0.46806* | | *TRUE* |
| *UFD1L* | *1.19141* | *512* | *1* | *610* | *1* | *1* | *.* | *409* | *0* | *303* | *2* | *0.182132* | | *FALSE* |
| *ZDHHC8* | *0.65496* | *503* | *10* | *593* | *18* | *0.33911* | *0.48966* | *403* | *6* | *296* | *9* | *0.19405* | | *TRUE* |
| *ZNF74* | *1.07321* | *504* | *9* | *601* | *10* | *1* | *1.24587* | *404* | *5* | *302* | *3* | *1* | | *TRUE* |
|  |  |  |  |  |  |  |  |  |  |  |  |  | |  |
| *Functional variants included frame-shift insertions, frame-shift deletions, splicing, non-synonymous and nonsense SNPs. The analysis excluded additional subjects compared with the variant summary table 1 (e.g. subjects without available GWAS data). | | | | | | | | | | | |  | |  |

|  | **Table S3. Distribution of rare functional variants in TCF4 in schizophrenia cases and controls.** | | | | | | | | | |  |
| --- | --- | --- | --- | --- | --- | --- | --- | --- | --- | --- | --- |
| ***Sample Set*** | | **Chr** | **Position^a^** | **Alleles** | **Call Rate** | **Variant Type**^b^ | **# of Carriers in Cases** | **# of Carriers in Controls** | **a.a. change^c^** |  | **Conservation Score^d^** |
| ***Replication*** | | 18 | 52921795 | C_A | 100 | Missense | 1 | 0 | G428V |  | 5.62 |
|  |  | 18 | 52928743 | G_A | 100 | Missense | 2 | 0 | A315V |  | 5.98 |
|  |  | 18 | 52937089 | G_A | 100 | Missense | 1 | 0 | P299S |  | 5.8 |
|  |  | 18 | 53070851 | C_T | 100 | Splicing | 1 | 0 | … |  | 4.75 |
|  |  | 18 | 53128250 | T_A | 100 | Missense | 1 | 0 | S102C |  | 4.8 |
| ***Discovery*** | | 18 | 52928743 | G_A | 100 | Missense | 3 | 1 | A315V |  | 5.98 |
|  |  | 18 | 53018123 | G_A | 100 | Missense | 0 | 1 | L161F |  | 5.41 |
|  |  | 18 | 53128285 | T_C | 100 | Missense | 0 | 1 | N90S |  | 5.95 |
| ^a^. The sequencing reads were mapped to human genome assembly hg19. | | | | | | | |  |  |  |  |
| ^b^. The variants were annotated according to the ENSEMBL V63 transcripts. | | | | | | | |  |  |  |  |
| ^c^. Amino acid changes are based on transcript NM_001083962.  ^d^. Conservation score was derived from genomic evolutionary rate profiling (GERP). | | | | | | | |  |  |  |  |
